# PPARs in Alzheimer's Disease

**DOI:** 10.1155/2008/403896

**Published:** 2008-07-14

**Authors:** Markus P. Kummer, Michael T. Heneka

**Affiliations:** Department of Neurology, University of Bonn, Sigmund-Freud-Strasse 25, 53127 Bonn, Germany

## Abstract

Peroxisome proliferator-activated receptors (PPARs) are well studied for their peripheral physiological and pathological impact, but they also play an important role for the pathogenesis of various disorders of the central nervous system (CNS) like multiple sclerosis, amyotrophic lateral sclerosis, Alzheimer's, and Parkinson's disease. The observation that PPARs are able to suppress the inflammatory response in peripheral macrophages and in several models of human autoimmune diseases lead to the idea that PPARs might be beneficial for CNS disorders possessing an inflammatory component. The neuroinflammatory response during the course of Alzheimer's disease (AD) is triggered by the neurodegeneration and the deposition of the *β*-amyloid peptide in extracellular plaques. Nonsteroidal anti-inflammatory drugs (NSAIDs) have been considered to delay the onset and reduce the risk to develop Alzheimer's disease, while they also directly activate PPAR*γ*. This led to the hypothesis that NSAID protection in AD may be partly mediated by PPAR*γ*. Several lines of evidence have supported this hypothesis, using AD-related transgenic cellular and animal models. Stimulation of PPAR*γ* receptors by synthetic agonist (thiazolidinediones) inducing anti-inflammatory, anti-amyloidogenic, and insulin sensitising effects may account for the observed effects. Several clinical trials already revealed promising results using PPAR agonists, therefore PPARs represent an attractive therapeutic target for the treatment of AD.

## 1. INTRODUCTION

The peroxisome proliferator-activated receptors (PPARs) belong to the family of nuclear hormone receptors (NHR) that comprise 48 human ligand-inducible transcription factors, which activity is regulated by steroids and lipid metabolites. Three different PPAR genes (PPAR*α*, PPAR*β*, also called *δ*, and PPAR*γ*) have been identified in all metazoa, that show unique spatiotemporal tissue-dependent patterns of expression during fetal development in a variety of cell types deriving form the ecto-, meso- or endoderm in rodents. Functionally PPARs are involved in adipocyte differentiation, lipid storage, and glucose homeostasis of the adipose tissue, brain, placenta, and skin (reviewed in [1]).

### 1.1. Functions of PPARs

PPARs act principally as lipid sensors and regulate the whole body metabolism in response to dietary lipid intake and direct their subsequent metabolism and storage [2]. The prototypic member of the family, PPAR*α*, was initially reported to be induced by peroxisome proliferators, and now denotes the subfamily of three related receptors. The natural ligands of these receptors are dietary lipids and their metabolites. The specific ligands have been difficult to establish, owing to the relatively low affinity interactions and broad ligand specificity of the receptors.

PPAR*α* acts primarily to regulate energy homoeostasis through its ability to stimulate the breakdown of fatty acids and cholesterol, driving gluconeogenesis and reduction in serum triglyceride levels. This receptor acts as a lipid sensor, binding fatty acids and initiating their subsequent metabolism. PPAR*γ* binds a number of lipids including fatty acids, eicosanoids, and other natural lipid ligands. Its dominant action is to stimulate adipocyte differentiation and to direct lipid metabolites to be deposited in this tissue. PPAR*γ* operates at the critical metabolic intersection of lipid and carbohydrate metabolism. PPAR*γ* activation is linked to reduction in serum glucose levels, likely as a secondary effect of its ability to regulate endocrine factors. It is this latter activity that has led to the development of specific PPAR*γ* agonists for the treatment of type II diabetes [3]. The PPAR*β*/*δ* binds and responds to VLDL-derived fatty acids, eicosanoids including prostaglandin A1 [4] and appears to be primarily involved in fatty acid oxidation, particularly in muscle.

PPARs regulate gene expression by forming heterodimers with retinoid-X-receptors (RXRs). Stimulation of target gene expression is controlled by specific PPAR-response elements in the promoter region (PPREs). Under unstimulated conditions, these heterodimers are associated with corepressors, like N-CoR and SMRT, which suppress gene transcription [1]. Upon ligand binding to the nuclear receptor, the corepressors are displaced and transcriptional coactivators are recruited to the receptor. These coactivator receptor complexes finally induce the formation of a much larger transcriptional complex which subsequently links the basal transcriptional apparatus and initiates gene transcription. In addition, activity of PPARs is also regulated by posttranslational modification such as phosphorylation and sumoylation [5, 6].

Like other NHR, PPARs also inhibit proinflammatory gene expression by a controversial mechanism of *transcriptional transrepression,* which is not mediated by their binding to PPREs. PPAR*γ* is able to suppress expression of proinflammatory genes in myeloid lineage cells, such as microglia and macrophages, and in the vasculature [7], by suppressing the action of NF*κ*B, AP-1, and STAT1 transcription factors [8]. A mechanistic model for the PPAR*γ*-mediated transrepression has recently been proposed. NF*κ*B-regulated inflammatory genes are maintained under basal conditions in a repressed state by N-Cor containing corepressor complexes. Upon exposure to proinflammatory stimuli this complex is dismissed and gene expression is initiated. This dismissal can be prevented by sumoylated PPAR*γ*: PPAR*γ* agonist complexes that stabilizes NCor complexes at the promoters of NF*κ*B-regulated genes, thus preventing inflammatory gene expression [9, 10].

Binding of PPARs to their specific ligands leads to conformational changes which allow corepressor release and coactivator recruitment. Even though all PPARs can be attributed to a common ancestral nuclear receptor, each PPAR isotype has its own properties with regard to ligand binding. Synthetic thiazolidinediones (TZDs), which are commonly prescribed for the treatment of type II diabetes, are selective PPAR*γ* ligands. Naturally occurring PPAR*γ* ligands include eicosanoids and the prostaglandin 15d-PGJ2. The best characterized PPAR*γ* agonists are the TZDs including pioglitazone and rosiglitazone which are Food and Drug Administration (FDA) approved for treatment of type II diabetes and troglitazone, which was withdrawn in 2000. PPAR*α* ligands include fibrates that are commonly used for the treatment of hypertriglyceridemia and the synthetic agonists WY14,643, and GW7647. PPAR*β*/*δ* agonists include the prostacyclin PGI2, and synthetic agents including GW0742, GW501516, and GW7842. All three PPAR isotypes can be activated by polyunsaturated fatty acids with different affinities and efficiencies [11]. An overview addressing the affinity of several natural and synthetic ligands has recently been summarized [12].

### 1.2. PPARs during development

PPAR*α* and *γ* transcripts appear late during fetal development of rat and mouse (day 13.5 of gestation), with similar expression pattern to their adult distribution. PPAR*α* is found in the liver, the kidney, the intestine, the heart, the skeletal muscle, the adrenal gland, and the pancreas. PPAR*γ* expression is restricted to the brown adipose tissue (day 18.5 of gestation), and to the CNS (day 13.5 to 15.5 of gestation). Compared to the two other isotypes, PPAR*β*/*δ* is expressed ubiquitously and earlier during fetal development [13]. In adult rodent organs, the distribution of PPAR*α* is similar to its fetal pattern of expression.

Not much is known about the expression of the PPARs during human development [14–16]. PPAR*α* is most highly expressed in tissues that catabolise fatty acids, such as the adult liver, heart, kidney, large intestine, and skeletal muscle. PPAR*β*/*δ* mRNA is present ubiquitously, with a higher expression in the digestive tract and the placenta. PPAR*γ* is abundantly expressed in the white adipose tissue, and is present at lower levels in the skeletal muscle, the heart, and the liver. Surprisingly, and in contrast to rodents, human PPAR*γ* seems to be absent from lymphoid tissues, even though PPAR*γ* has been shown to be present in macrophages in human atheroma.

### 1.3. PPARs in the brain

All three PPAR isotypes are coexpressed in the nervous system during late rat embryogenesis, and PPAR*β*/*δ* is the prevalent isotype. The expression of the three PPAR isotypes peaks in the rat CNS between day 13.5. and 18.5 of gestation. Whereas PPAR*β*/*δ* remains highly expressed in this tissue, the expression of PPAR*α* and *γ* decreases postnatally in the brain [17]. While PPAR*β*/*δ* has been found in neurons of numerous brain areas, PPAR*α* and *γ* have been localized to more restricted brain areas [18, 19]. Analysis of the expression of PPARs in different brain regions of adult mice revealed that PPAR*β*/*δ* mRNAs are preferentially found in the cerebellum, the brain stem, and the cortex, whereas PPAR*γ* mRNAs are enriched in the olfactory areas as well as in the cortex. Expression of all three isotypes was found to be low to moderate in the hippocampus. More detailed analysis of PPARs expression within the hippocampus by in situ hybridisation revealed a ubiquitous expression pattern for PPAR*α*, whereas PPAR*β* was found to be enriched in the dentate gyrus/CA1 region and PPAR*γ* expression was restricted to the CA3 region [20].

Even though this pattern of expression, which is isotype specific and regulated during development, suggests that the PPARs may play a role during the formation of the CNS, their function in this tissue are still poorly understood. Both in vitro and in vivo observations show that PPAR*β*/*δ* is the prevalent isoform in the brain, and is found in all cell types, whereas PPAR*α* is expressed at very low levels predominantly in astrocytes [21]. Acyl-CoA synthetase 2, which is crucial in fatty acid utilization, is regulated by PPAR*β*/*δ* at the transcriptional level, providing a facile measure of PPAR*β*/*δ* action. This observation strongly suggests that PPAR*β*/*δ* participates in the regulation of lipid metabolism in the brain. This hypothesis is further supported by the observation that PPAR*β*/*δ* null mice exhibit an altered myelination of the corpus callosum. Such a defect was not observed in other regions of the central nervous system, and the expression of mRNA encoding proteins involved in the myelination process remained unchanged in the brain.

Expression of all PPAR isoforms, including PPAR*γ*, has been confirmed in the adult brain. Furthermore, it has been suggested that PPAR activation in neurons may directly influence neuron cell viability and differentiation [22–26]. The localization of PPARs has also been investigated in purified cultures of neural cells. PPAR*β*/*δ* is expressed in immature oligodendrocytes and its activation promotes differentiation, myelin maturation, and turnover [27, 28]. The PPAR*γ* is the dominant isoform in microglia. Astrocytes possess all three PPAR isotypes, although to different degrees depending on the brain area and animal age [29, 30]. The role of PPARs in the CNS is mainly been related to lipid metabolism, however, these receptors, especially PPAR*γ*, have been implicated in neural cell differentiation and death as well as in inflammation and neurodegeneration [23]. PPAR*α* has been suggested to be involved in the acetylcholine metabolism [31] and to be related to excitatory amino acid neurotransmission and oxidative stress defence [18].

## 2. INFLAMMATION AND ALZHEIMER's DISEASE

**Figure 1:**
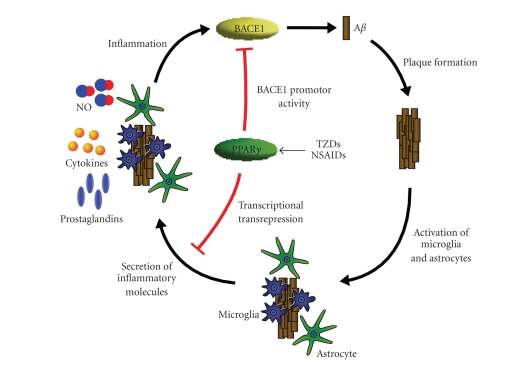
Effects of PPAR*γ* on A*β* metabolism. Excessive production or insufficient clearance of A*β* results in its aggregation and finally in the formation of amyloid plaques. This process induces the activation of microglia as well as astrocytes which respond with the secretion of proinflammatory molecules like NO, cytokines, and prostaglandins developing the inflammatory phenotype of AD. In addition, cytokines are able to increase BACE1 activity thereby stimulating A*β* production. PPAR*γ* agonists are able to abate both effects by either transrepress the production of proinflammatory molecules or directly interfere with the binding of PPAR*γ* to a PPRE in the BACE1 gene promoter.

The number of individuals with the Alzheimer's disease (AD) is dramatically increasing throughout the developed world. The large number of affected individuals and the increasing prevalence of the disease presents a substantial challenge to health care systems and does so in the face of substantial economic costs. The pathological hallmarks of AD are the formation of extracellular plaques consisting of amyloid-*β* peptides and intracellular neurofibrillary tangles made up from hyperphosphorylated tau protein, causing neuronal death that is responsible for progressive memory loss and inexorable decline of cognitive functions [32, 33]. Analysis of the genetic forms and animal models suggested a pivotal role for the amyloid *β* peptide (A*β*), nevertheless, the biological basis of AD, especially of the sporadic forms, is still poorly understood. Genetically, A*β* metabolism is closely linked to lipid metabolism as a certain allele of the lipid carrier protein ApoE is associated with significantly increased risk for AD [34]. Another key hallmark of AD brain is the presence of chronic neuroinflammation without any signs of leukocyte infiltration. Amyloid plaques within the brain are populated by abundant, activated microglia, and astrocytes [35]. Microglial activation is accompanied by the secretion of inflammatory cytokines and chemokines including interleukin (IL)-1*β*, IL-6, monocyte chemotactic protein-1, (MCP-1), and tumor necrosis factor (TNF)-*α* [36]. It was posited that activation of microglia and the concurrent production of inflammatory molecules may deteriorate and accelerate the progression of AD and therefore the neuronal loss [35]. Neuronal expression of inflammatory enzyme systems, including iNOS, has also been described in AD [37–39]. Altogether, these data suggest that anti-inflammatory therapies may be beneficial for AD treatment (see Figure 1).

## 3. EFFECTS OF PPAR*γ* AGONISTS ON ALZHEIMER's DISEASE

PPAR*γ* is expressed in the brain at the low levels under physiological conditions. Recently, a detailed gene expression analysis has demonstrated that mRNA levels are elevated in AD patients [40]. This suggests that PPAR*γ* plays a role in the modulation of the pathophysiology of AD. Currently used drugs are mainly targeted at symptomatic improvement of the patients. These agents have only modest therapeutic efficacy over rather short periods. Thus, the development of new therapeutic approaches is of critical importance.

The initial studies exploring the actions of PPAR*γ* in AD were based on the ability of nonsteroidal anti-inflammatory drugs (NSAID) to activate this receptor. A number of epidemiological studies demonstrated that NSAID treatment reduces AD risk by as much as 80% and it was suggested that these effects arise from the ability of these drugs to stimulate PPAR*γ* and to inhibit inflammatory responses in the AD brain [41–45]. This hypothesis is supported by the finding that experimental expression of iNOS in neurons resulted in time-dependent neuronal cell death which was prevented by activation of PPAR*γ* in vitro and in vivo [23, 46]. In addition, PPAR*γ* activation in microglial cells suppressed inflammatory cytokine expression, iNOS expression, and NO production as well as inhibited COX2 and therefore the generation of prostanoids [47]. These latter effects result from the ability of PPAR*γ* to suppress proinflammatory genes through antagonism of the transcription factor NF*κ*B, (and to a lesser extent, AP-1 and STATs) [8]. PPAR*γ* agonists have also been demonstrated to suppress the A*β*-mediated activation of microglia in vitro and prevented cortical or hippocampal neuronal cell death [47–49]. In a rat model of cortical A*β* injection, coinjection of ciglitazone and ibuprofen or oral pioglitazone administration potently suppressed A*β*-evoked microglial cytokine generation. The effects of the PPAR*γ* agonists pioglitazone and ibuprofen have been investigated in animal models of AD (Tg2576) that overexpress human APP. Pioglitazone was selected as it passes the blood brain barrier, although with limited penetration [50]. 12 months old Tg2576 mice were treated orally for 4 months resulting in a significant reduction of SDS-soluble A*β*40. A*β*42 levels were only significantly lowered for ibuprofen-treated animals, but a trend was observed for pioglitazone [51].

The modest effects of pioglitazone in this study were thought to be due to poor drug penetration into the brain. In a subsequent study treatment with larger doses of pioglitazone in aged APPV717I transgenic mice significantly decreased microglial and astroglial activation as well as A*β* plaque burden [52]. The finding that PPAR*γ* agonists elicited a reduction in amyloid pathology may be the result of the ability of PPAR*γ* to affect A*β* homeostasis. According to this hypothesis, evidence has been provided that immunostimulated Β-site APP cleaving enzyme (BACE1) expression is silenced by a PPAR*γ*-dependent regulation of the BACE1 gene promoter [53, 54]. Similarly, oral pioglitazone treatment of APP transgenic mice reduced BACE1 transcription and expression. A recent study has found that PPAR*γ* is associated with enhanced A*β* clearance. PPAR*γ* activation, in both glia and neurons, led to a rapid and robust uptake and clearance of A*β* from the medium [55]. It has also been suggested that NSAIDs act directly on A*β* processing by the *γ*-secretase complex resulting in selective decrease of A*β*42 production [56, 57], even so this hypothesis has recently been challenged [58, 59].

Additionally, modulation of the Wnt/*β*-catenin signalling pathway may also account for some PPAR*γ*-mediated beneficial effects in AD since recent findings show that PPAR*γ*-mediated protection of hippocampal neurons against A*β*-induced toxicity directly correlates with *β*-catenin levels, inhibition of GSK-3*β* activity, and increased levels of Wnt-target genes [24, 60]. Furthermore, recent evidence suggests that PPAR*γ* activation may also provide protection from excitotoxic stimuli [61] and positively influences neural stem cell proliferation and differentiation [62], both mechanisms that could potentially influence the overall salutary effects observed in models of neurodegenerative disease.

In a further animal study, Pedersen and colleagues have demonstrated that rosiglitazone treatment of Tg2576 mice resulted in behavioural improvement in these animals as well as in reduction of A*β*42 in the brain. Treatment with rosiglitazone for 34 months enhanced spatial working and reference memory [63]. Significantly, drug treatment was associated with a 25% reduction in A*β*1-42 levels, however A*β*1-40 levels remained unchanged. This reduction of A*β*1-42 was argued to arise from increased levels of insulin degrading enzyme (IDE) in rosiglitazone-treated transgenic mice, even so IDE has not been reported to be regulated by PPAR*γ*. IDE is an A*β* degrading metalloprotease that has been genetically linked to AD [64].

The outcome of two clinical trials of the PPAR*γ* agonist rosiglitazone has recently been reported [65, 66]. These studies reported that rosiglitazone therapy improves cognition in a subset of AD patients. Rosiglitazone does not pass the blood-brain barrier [65, 66], and this has been a confound in interpreting the CNS actions resulting from the administration of this drug. These data were interpreted as evidence for a significant role for peripheral insulin sensitivity in cognition. AD risk and memory impairment is associated with hyperinsulinemia, and insulin resistance, features which characterize type II diabetes [65, 67]. Indeed, type II diabetes is associated with increased risk of AD [67, 68]. Indeed, in a replication study PPAR*γ* was found to be significantly associated with Alzheimer's disease [69]. Likewise, the Pro12Ala polymorphism within the exon 2 of PPAR*γ* has been already linked to type 2 diabetes, insulin sensitivity, obesity, and cardiovascular diseases (for review see [70]). Even so the effect of this polymorphism is heterogeneous, since the Pro12Ala variant is associated with a reduced risk for diabetes [71–73], it has recently been shown that this polymorphism is associated with higher risk for Alzheimer's disease in octogenarians even after adjustment for the ApoE4 allele [74].

Clinical investigations of insulin-sensitizing TZDs that are in clinical use for type II diabetes are currently ongoing. A small study of 30 patients with mild AD or MCI found that 6 months of treatment with rosiglitazone resulted in improved memory and selective attention. A larger trial of rosiglitazone in AD patients has recently been reported [75]. More than 500 patients with mild to moderate AD were treated for 6 months with rosiglitazone, resulting in a statistically significant improvement in cognition in those patients that did not possess an ApoE4 allele [65]. Patients with ApoE4 did not respond to the drug and showed no improvement in standard cognitive tests. As an explanation it was suggested that rosiglitazone acts on mitochondria in the brain, increasing their metabolic efficiency and number. This hypothesis is supported by the observation that rosiglitazone induces neuronal mitochondrial DNA expression, enhances glucose utilization by inducing transcription of glucose metabolism and mitochondrial biogenesis genes leading to improved cellular function in mice. Noteworthy, these effects where also observed in animals expressing the ApoE4 allele. Determination of the amount of rosiglitazone in the brain revealed that 9–14% of the blood rosiglitazone crossed the blood brain barrier after oral treatment [76]. The actions of TZDs on mitochondria occur through both PPAR*γ*-dependent and independent mechanisms [77]. The basis of the differential effects of rosiglitazone in individuals depending on their ApoE genotype is unexplained. The outcome of this clinical trial is, however, consistent with previous findings with respect to the influence of the ApoE4 genotype [78–80].

## 4. CONCLUSION

PPARs exhibit a wide range of activities to positively influence the pathology of Alzheimer's disease. Beside the ameliorating effect of PPAR*γ* agonists on the inflammatory status of the AD brain by repressing the secretion of proinflammatory molecules and the enhancement of mitochondrial function, a direct involvement in the processing of the A*β* peptide has been demonstrated (Figure 1). The compelling results from animal models of Alzheimer's disease underline the beneficial effects of PPAR agonists for future therapies. The importance of these activities for the disease altering actions of PPAR agonist as well as the underlying molecular mechanisms have to be elucidated in ongoing and future research.
